# PET/CT molecular imaging in the era of immune-checkpoint inhibitors therapy

**DOI:** 10.3389/fimmu.2022.1049043

**Published:** 2022-10-20

**Authors:** Yuan Gao, Caixia Wu, Xueqi Chen, Linlin Ma, Xi Zhang, Jinzhi Chen, Xuhe Liao, Meng Liu

**Affiliations:** Department of Nuclear Medicine, Peking University First Hospital, Beijing, China

**Keywords:** positron emission tomography/computed tomography (PET/CT), molecular imaging, immune-checkpoint inhibitors (ICIs), tumor microenvironment (TME), metabolic parameter, molecular probe

## Abstract

Cancer immunotherapy, especially immune-checkpoint inhibitors (ICIs), has paved a new way for the treatment of many types of malignancies, particularly advanced-stage cancers. Accumulating evidence suggests that as a molecular imaging modality, positron emission tomography/computed tomography (PET/CT) can play a vital role in the management of ICIs therapy by using different molecular probes and metabolic parameters. In this review, we will provide a comprehensive overview of the clinical data to support the importance of ^18^F-fluorodeoxyglucose PET/CT (^18^F-FDG PET/CT) imaging in the treatment of ICIs, including the evaluation of the tumor microenvironment, discovery of immune-related adverse events, evaluation of therapeutic efficacy, and prediction of therapeutic prognosis. We also discuss perspectives on the development direction of ^18^F-FDG PET/CT imaging, with a particular emphasis on possible challenges in the future. In addition, we summarize the researches on novel PET molecular probes that are expected to potentially promote the precise application of ICIs.

## Introduction

Cancer immunotherapy has paved a new way for the treatment of many types of malignancies, particularly advanced-stage cancers, by intervening in the abnormal immune processes, reshaping the tumor microenvironment (TME), and restoring immune surveillance (1). Immune-checkpoint inhibitors (ICIs), such as the blocking antibodies of programmed cell death protein-1 (PD-1), programmed death-ligand 1 (PD-L1), and cytotoxic T lymphocyte-associated protein 4 (CTLA-4), have brought considerable clinical benefits to cancer patients. However, only a subset of patients can benefit from ICIs therapies, and some might even experience severe immune-related adverse events (irAEs) and detrimental hyperprogressive disease (2). Emerging preclinical and clinical evidence indicates that the reciprocity between ICIs and TME may play a complex and important influence on ICIs therapy, but the specific mechanism is still unclear. How to characterize TME noninvasively and effectively, so as to deeply elucidate its potential mechanisms in immunotherapy and precisely guide the use of ICIs, is continually attracting research interest worldwide.

It is well-known that positron emission tomography (PET)/computed tomography (CT) can reflect the biological information of the living body noninvasively and dynamically by using different kinds of imaging agents. Fluorine-18 fluorodeoxyglucose (^18^F-FDG), the most commonly used PET/CT imaging agent, has been increasingly applied in the immunotherapeutic management. It can reflect the level of glucose accumulation in both primary tumor tissues and metastatic lesions by tracking glucose uptake through a single scan. The metabolic parameters obtained from ^18^F-FDG PET/CT arguably provide useful indications of the tumor burden (3). Accumulating evidence suggests that ^18^F-FDG PET/CT imaging can play a vital role in ICIs therapy, including TME characterization, irAEs assessment, efficacy evaluation, prognosis prediction, and so on.

In this review, we focus on the characteristics of TME associated with immunotherapy, and provide an overview of the clinical data associated with the application of ^18^F-FDG PET/CT imaging in the treatment of ICIs. Furthermore, we discuss perspectives on the development direction of ^18^F-FDG PET/CT imaging, with a particular emphasis on possible challenges in the future. We also summarize the researches on novel PET/CT molecular imaging, which may potentially promote the precise application of ICIs.

## Characteristics and classifications of TME

Compared with traditional treatments, such as radiotherapy and chemotherapy, ICIs treatment is more closely related to TME. The efficacy of ICIs may be influenced by various mechanisms related to the tumor or the host, among which TME is being widely investigated as a critical factor. The characteristics of TME vary in different individuals and cancer types, which will affect the immune response to ICIs treatment.

### Compositions and metabolism of TME

**Figure 1:**
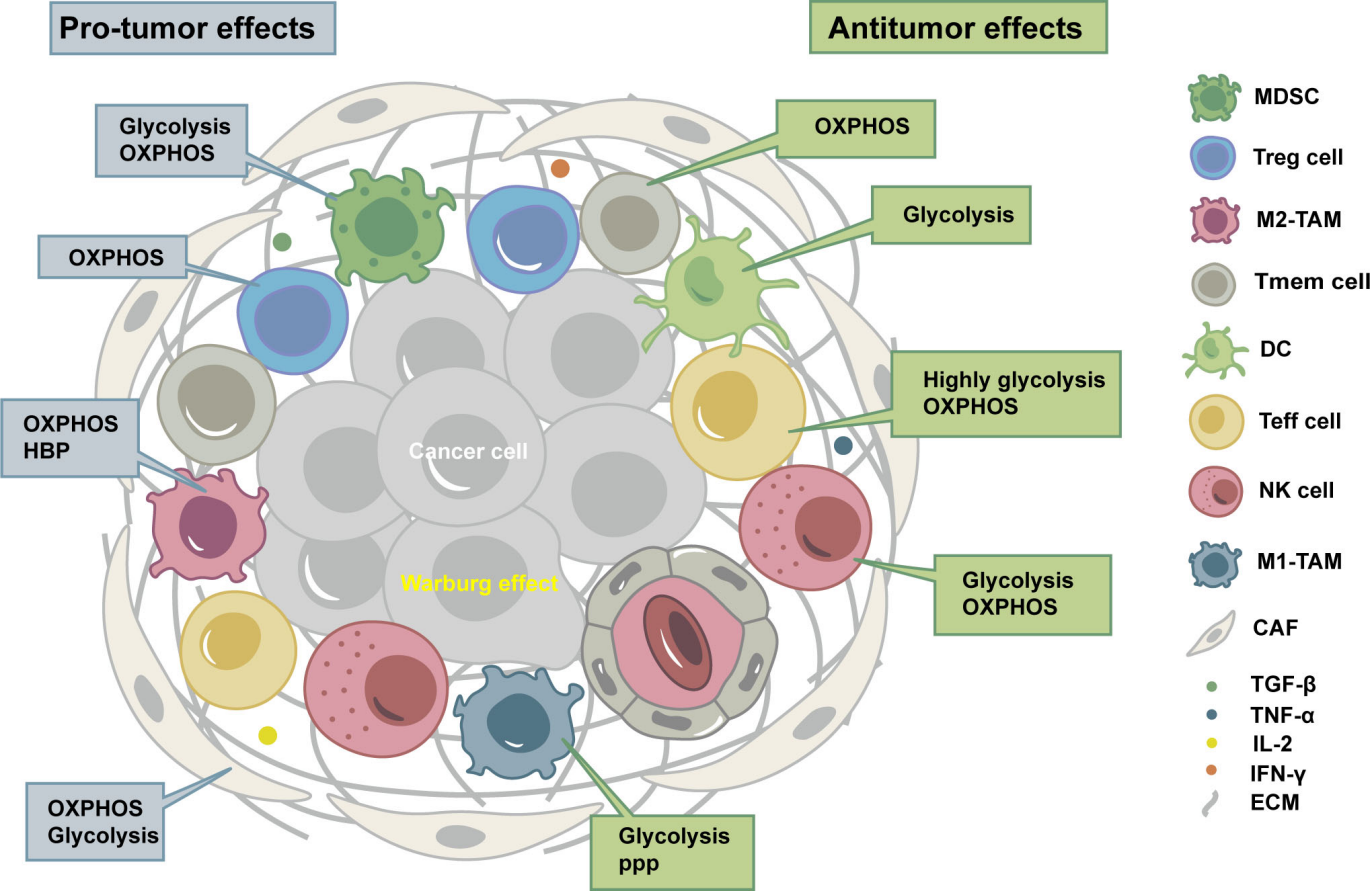
Tumor microenvironment (TME) is composed of tumor cells, immune cells, stromal cells, extracellular matrix, and exosomes, thus forming a microenvironment with the characteristics of inflammation, hypoxia, acidity, and immunosuppression. Different types of cells in TME have their preferred metabolic phenotypes. OXPHOS, oxidative phosphorylation; HBP, hexosamine biosynthesis pathway; PPP, pentose phosphate pathway; MDSC, myeloid-derived suppressor cell; Treg cell, regulatory T cell; M2-TAM, immunosuppressive macrophages; Tmem cell, CD8^+^ memory T cells; DC, dendritic cell; Teff cell, CD8^+^ effector T cells; NK cell, natural killer cell; M1-TAM, inflammatory tumor-associated macrophages; CAF, cancer-associated fibroblast; TGF-β, transforming growth factor-β; TNF-α, tumor necrosis factor-α; IL-2, Interleukin-2; IFN-γ, Interferon-γ; ECM, extracellular matrix.

TME is composed of tumor cells, immune cells, stromal cells, extracellular matrix, and exosomes (4), thus forming a microenvironment with the characteristics of inflammation, hypoxia, acidity, and immunosuppression. Different types of cells in TME have their preferred metabolic phenotypes (**Figure 1**).

Tumor cells generally experience metabolic reprogramming, especially glucose metabolism, to adapt to immunosuppressive TME. Even under aerobic conditions, tumor cells are typically characterized by glycolysis, resulting in high rates of glucose intake with high lactate excretion, which is known as the Warburg effect (5). There are fundamental differences between the metabolic programs of cancer cells and immune cells, as well as between different immune cells (6).

Immune cells can be divided into immune-activating cells and immunosuppressive cells. The characteristics of TME might inhibit antitumor immune cells and lead to their exhaustion or senescence (7), but tumor-promoting immune cells mostly show tolerance (8). Immune-activating cells include CD8^+^ effector T (Teff) cells, CD8^+^ memory T (Tmem) cells, CD4^+^ T helper 1 (Th 1) cells, dendritic cells (DCs), natural killer (NK) cells, inflammatory tumor-associated macrophages (M1-TAM), B cells, and neutrophils. Immunosuppressive cells include regulatory T (Treg) cells, myeloid-derived suppressor cells (MDSCs), and immunosuppressive macrophages (M2-TAM), which subvert antitumor immunity by secreting cytokines or interfering with metabolism (5).

Stromal cells include cancer-associated fibroblasts (CAFs), endothelial cells, and pericytes. Like immune cells, stromal cells could interact with tumor cells, modulate their metabolic behavior, and contribute to migration, invasion, and evasion of immune surveillance (9). CAFs can carry out aerobic glycolysis and secrete lactate and pyruvate as fuels for neighboring tumor cells. A metabolic cross-talk exists between tumor cells and CAFs, referred to as a reverse-Warburg effect. CAFs are also characterized by increased synthesis and secretion of glutamine, which is consumed by cancer cells, thus allowing them to sustain nucleotide generation and oxidative phosphorylation (OXPHOS) to obtain high proliferation (10).

### Classifications of TME associated with ICIs therapy

With the continuous understanding of the dynamic interaction (promote or hinder) between the status of TME and the treatment with ICIs, several TME classifications based on immunotherapeutic rationale have been proposed. These types are associated with therapy response and might be helpful in selecting the appropriate immunotherapy strategy and suitable patients.

Tumor immune microenvironment (TIME) refers to the immunological characteristics of TME, mainly including the cell types, infiltration degrees, and molecular expression levels of immune cells. Based on the degree and location of tumor-infiltrating lymphocytes (TILs) in TIME, tumors can be classified into “cold” or “hot” ones, or more precisely, divided into three types of immune-inflamed, immune-excluded, and immune-desert (11). In addition, PD-L1 expression and TILs infiltration are essential features of TIME related to ICIs (12, 13). Hence, TIME can be divided into four tumor immune microenvironment types (TIMTs) according to these two characteristics, i.e., PD-L1−/TIL−, PD-L1+/TIL+, PD-L1−/TIL+, and PD-L1+/TIL− (14).

Considering that the metabolic and immune characteristics of TME are important theoretical bases for tumor immunotherapy, Siska et al. recommended the metabolic-tumor-stroma score (MeTS) to describe the characteristics of tumor metabolism and cell heterogeneity (15): (1) OXPHOS metabolic type and high T cell infiltration; (2) reverse Warburg type; (3) mixed type; and (4) Warburg type and low T cell infiltration.

## Application of ^18^F-FDG PET/CT imaging in ICIs treatment

**Figure 2:**
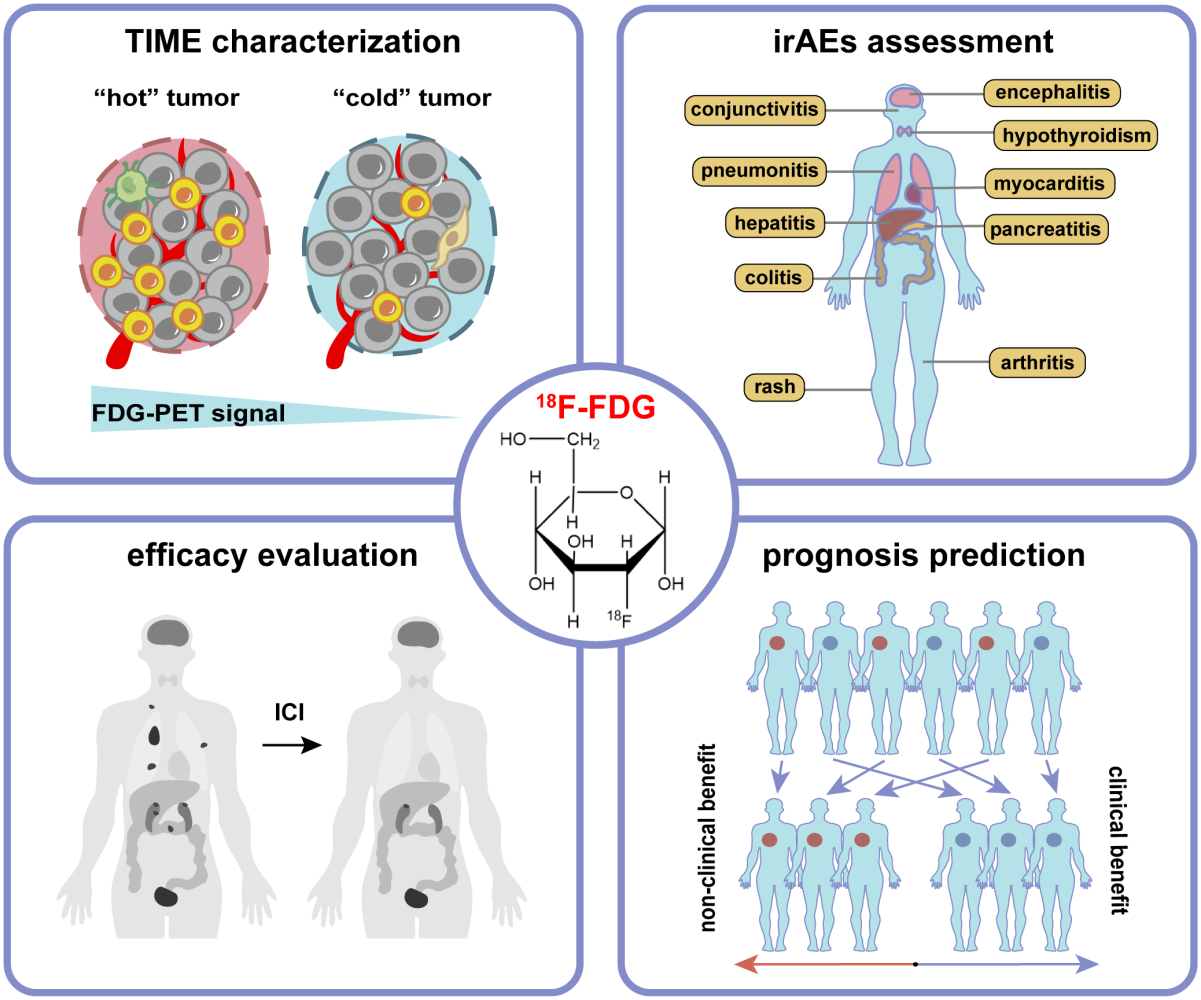
The application of ^18^F-FDG PET/CT imaging in tumor immunotherapy includes characterization of tumor immune microenvironment (TIME), assessment of immune-related adverse events (irAEs), evaluation of therapeutic efficacy, and prediction of prognosis.

Due to the Warburg effect, tumor cells are usually characterized by high glucose metabolism, i.e., increased FDG uptake is often induced in the case of over-expression of glucose transporters (GLUT), such as GLUT1 and GLUT3. A set of studies have shown that GLUT1 expression is correlated with tumor size and hypoxia of TME, and the latter activates hypoxia-inducible factor 1-alpha (HIF-1α) to trigger the Warburg effect and upregulate GLUT expression (16). It has also been elucidated that HIF-1α could directly bind to the hypoxia response element in the PD-L1 proximal promoter and control its expression under hypoxic conditions (17). Besides, activation of some immune cells, including CD4^+^ and CD8^+^ T cells, is accompanied by increased metabolism, such as upregulated aerobic glycolysis, tricarboxylic acid (TCA) cycle, and OXPHOS (6). The above mechanisms may provide a theoretical basis for the role of ^18^F-FDG PET/CT imaging in ICIs therapy, including characterization of TIME, assessment of irAEs, evaluation of therapeutic efficacy, and prediction of prognosis (**Figure 2**).

### Characterization of TIME

Delineation of TIME characteristics can help treatment formulation, efficacy evaluation, and prognosis prediction (18). Due to the complexity of TIME components and their dynamic changes during the treatment process of ICIs, traditional methods such as biopsy have limitations in reflecting TIME. As a non-invasive and functional whole-body imaging modality, ^18^F-FDG PET/CT imaging has some potential advantages in characterizing the overall glucose metabolism of tumor cells, activated immune cells, and stromal cells in the TME of the primary lesions.

The majority of researches on the application of ^18^F-FDG PET/CT imaging on TIME characterization focused on non-small-cell lung cancer (NSCLC) (**Table 1**). Zhao et al. carried out two studies with the largest sample sizes (419 cases and 428 cases) to investigate the relationship between PD-L1 expression and ^18^F-FDG uptake, using 22C3 and SP142 assays, respectively (19, 20). They both showed that maximum standardized uptake value (SUVmax) was significantly associated with PD-L1 expression in NSCLC. A meta-analysis across seven studies (473 patients) showed that the predictive sensitivity of the SUVmax for the expression of PD-L1 in NSCLC patients was 75%, and the specificity was 73% (41). Other metabolic parameters, such as mean standardized uptake value (SUVmean) and the ratio of metabolic to morphological lesion volumes (MMVR), have been reported to be correlated with PD-1 or PD-L1 expression (26, 27). ^18^F-FDG PET/CT radiomics have also been used to explore predictive models based on images and clinical information for PD-L1 expression (28–30). Additionally, Mitchell et al. found that high SUVmax was associated with reduced CD57^+^ cell density and increased T cell exhaustion gene signature (21). Wang et al. revealed that high SUVmax was associated with high infiltration of CD8^+^ T cells, M2 macrophages, and Foxp3^+^ Treg cells (22).

**Table 1:** Tumor microenvironment evaluation by ^18^F-FDG PET/CT imaging.

Histology	Parameters	Conclusions	References
NSCLC	SUVmax	positively associated with PD-L1 expression	(19–25)
		positively associated with CD8^+^ T cells, CD163^+^ TAMs and Foxp3+ Treg cells; negatively associated with CD57^+^ cells	(21, 22, 26)
	SUVmean	positively associated with PD-1 expression	(26)
	MMVR	negatively correlated with PD-L1 expression in TCs	(27)
	radiomics	models based on radiomics and/or clinicopathological characteristics showed good accuracy in predicting PD-L1 expression level	(28–30)
		showed good performance in predicting PD-L1^+^/TIL^+^ tumors	(31)
ccRCC	SUVmax	positively associated with PD-L1^+^/TIL^+^ and PD-L1^-^/TIL^+^ tumors	(32)
breast cancer	SUVmax	positively associated with TIL levels	(33–35)
		positively associated with PD-L1 expression	(33)
gastric cancer	SUVmax	positively correlated with CD3^+^ and Foxp3^+^ T cell counts	(36)
colorectal cancer	SUVmax, MTV, TLG	positively associated with PD-L1 expression	(37)
bladder cancer	SUVmax	positively associated with PD-L1 and PD-1 expression	(38)
nasopharyngeal carcinoma	SUVmax	negatively correlated with PD-L1 expression in TIICs and positively associated with PD-L1 expression in TCs	(39)
oral squamous cell carcinoma	SUVmax	negatively correlated with cold tumors (low tumoral PD-L1 and low stromal CD8^+^TILs)	(40)

NSCLC, non-small cell lung carcinoma; ccRCC, clear cell renal cell carcinoma; TCs, tumor cells; TIICs, tumor-infiltrating immune cells; TME, tumor microenvironment; TIMT, tumor immune microenvironment type; TIL, tumor-infiltrating lymphocyte; PD-L1, programmed death-ligand 1; PD-1, programmed cell death protein-1; SUVmax, maximum standardized uptake value; SUVmean, mean standardized uptake value; MTV, metabolic tumor volume; TLG, total lesion glycolysis; MMVR, ratio of metabolic to morphological lesion volumes.

In terms of TIMTs, Zhou et al. used dual-phase ^18^F-FDG PET/CT imaging to reflect metabolic dynamics in NSCLC and constructed a model combined metabolic signature (Meta-Sig) and clinical factors to predict PD-L1^+^/TIL^+^ tumors (AUC: 0.869, sensitivity: 77.27%, specificity: 82.61%) (42). Wu et al. analyzed the correlation between SUVmax and TIMT classification in patients with cell clear renal cell carcinoma (ccRCC) and suggested that SUVmax might be used as an indicator for TIMTs and thus help guide the treatment with ICIs (32).

In addition, the correlation between ^18^F-FDG PET/CT imaging and TIME in breast cancer, gastric cancer, colorectal cancer, bladder cancer, nasopharyngeal cancer, and oral squamous cell carcinoma has also been observed (**Table 1**).

### Assessment of irAEs

The perturbation of ICIs on the balance of the immune system can lead to a loss of self-tolerance and excessive immune activation of normal tissues, resulting in irAEs (43). The irAEs can affect nearly all organ systems, such as the neurologic, pulmonary, cardiovascular, gastrointestinal, endocrine, genitourinary, integumentary, skeletal and joint systems, and so on (44, 45). The irAEs resulting from different ICIs may vary. A systematic review found that deaths from CTLA-4 inhibitors were mainly caused by the irAEs of colitis (70%), and deaths from PD-1/PD-L1 inhibitors were mainly pneumonia (35%), hepatitis (22%), and neurotoxicity (15%) (46). For different types of tumors, the most commonly affected sites of irAEs are also different. For example, patients with NSCLC mainly show endocrine system and skin irAEs, and patients with melanoma mostly involve the skin and liver, while irAEs occur in patients with RCC are more common in the skin and gastrointestinal tract (47).

A number of current reports support that some irAEs seem to be associated with improved tumor response and better survival (48). This association may stay robust in certain cancer types (NSCLC, melanoma, RCC, and advanced urothelial cancer) and organ-specific irAEs (the skin and endocrine system) (49–52). But some reports pointed out that grade 3-5 irAEs (severe irAEs) are not associated with increased overall survival (OS) and progression-free survival (PFS) in NSCLC patients (53, 54). Oncologists should weigh the risk of irAEs against the benefit of ICIs before immunotherapy and take appropriate management once irAEs occur.

Therefore, it is necessary to judge the appearances of irAEs timely by noninvasive imaging methods. CT or magnetic resonance imaging (MRI) has been widely used to detect irAEs, especially in the lung, pancreas, liver, and nervous system (55). However, the utility of ^18^F-FDG PET/CT imaging in irAEs screening and monitoring is largely under-recognized currently (2).

**Figure 3:**
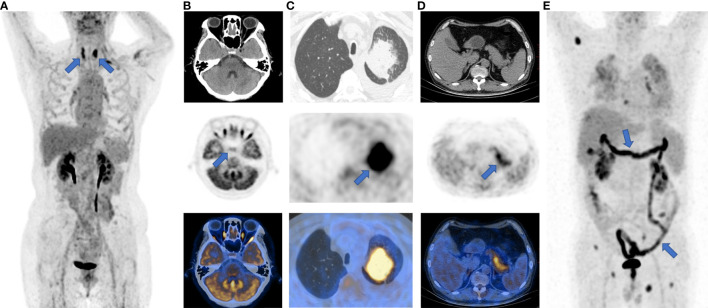
Typical images of irAEs in patients with ICI treatment. **(A)**, thyroiditis; **(B)**, hypophysitis; **(C)**, pneumonia; **(D)**, pancreatitis; **(E)**, enteritis. The sites of irAEs were marked with blue arrows on maximum intensity projection (MIP) and PET images.

^18^F-FDG PET/CT imaging may be a sensitive method to identify the development and severity of irAEs, which usually present as a new non-neoplastic lesion with increased FDG accumulation after ICI treatment (56). For instance, elevated thyroid SUVmax commonly suggests ICI-related thyroiditis (57), while a diffuse increase of FDG uptake in the pancreas is a characteristic manifestation of ICI-related pancreatitis (58). A study on patients with metastatic melanoma treated with ICIs found that novel quantitative imaging biomarkers, i.e. the SUV percentiles (SUV_X%_) of ^18^F-FDG uptake within the target organs, could be predictive of irAEs in the bowel, stomach, and thyroid (56). This study also demonstrated that some irAEs could be detected on ^18^F-FDG PET/CT imaging before the onset of clinical symptoms, which showed increased ^18^F-FDG uptake in the affected organs. The typical ^18^F-FDG PET/CT manifestations of irAEs can be seen in **Figure 3**.

### Evaluation of therapeutic efficacy

ICIs treatment can achieve antitumor effects by eliminating immunosuppression and reinvigorating Tmem cells, which are good for the patient’s long-term survival. However, ICIs can also lead to atypical response patterns, including pseudoprogression, hyperprogression, and mixed response.

**Figure 4:**
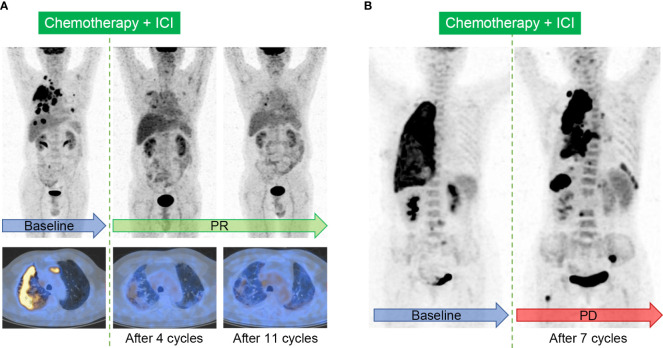
Typical cases evaluated by Response Evaluation Criteria in Solid Tumors (RECIST) 1.1 in patients with ICI treatment. **(A)**, Immunotherapy response in a 64-year-old male patient with right lung adenocarcinoma. The baseline image shows intensive FDG uptake in the primary tumor, accompanied with multifocal intrapulmonary metastasis, lymphadenopathy, and the involvement of pleura. Follow-up images after 4 cycles and sequential 11 cycles of the combination of chemotherapy and ICI show partial response (PR). **(B)**, Immunotherapy response in a 60-year-old female patient with right lung adenocarcinoma. Image after 7 cycles of the combination of chemotherapy and ICI shows the enlargement and increased metabolism in the primary tumor, and the onset of multiple new lesions in the lung, pleura, lymph nodes, liver, and bone, indicating progressive disease (PD).

In order to standardize the imaging evaluation of tumor treatment efficacy, a series of tumor treatment response evaluation criteria have been proposed. The CT-based evaluation criteria, i.e. Response Evaluation Criteria in Solid Tumors version 1.1 (RECIST 1.1) (59), were initially used directly. The typical cases evaluated by RECIST 1.1 were displayed in **Figure 4**. RECIST 1.1 was later adjusted for better ICI response evaluation. The modified RECIST 1.1 for immune-based therapeutics, i.e. iRECIST, classify the initial discovery of the suspected progression as initially unconfirmed progressive disease (iUPD) (60). Immune-modified RECIST (imRECIST) includes the measurable new lesions in the total tumor burden (61).

Meanwhile, the ^18^F-FDG PET (PET/CT)-based evaluation criteria have also been successively proposed (**Table 2**). The European Organization for Research and Treatment of Cancer 1999 criteria (EORTC) is the first metabolism criterion using SUVmax changes to determine antitumor treatment response (62). In 2009, Wahl et al. proposed the PET Response Criteria in Solid Tumors (PERCIST) (63), which has also been modified further. Immune PERCIST (iPERCIST) introduced the concept of unconfirmed progressive metabolic disease (UPMD) (64). Since immunotherapy may induce new inflammatory lesions that are detectable on ^18^F-FDG PET/CT, immunotherapy-modified response classification (imPERCIST5) was introduced. According to imPERCIST5, progressive metabolic disease (PMD) was defined only by an increase of the sum of peak standardized uptake values normalized for body lean body mass (SULpeak) by 30% (65). In 2018, Anwar et al. proposed PET Response Evaluation Criteria for Immunotherapy (PERCIMT) to evaluate clinical benefit based on the number and size of new lesions (66). According to PERCIMT, 4 or more new lesions (regardless of size), or 3 or more new lesions (diameter > 1 cm), or 2 or more new lesions (diameter > 1.5 cm), are all defined as no-clinical benefit, while the other cases are considered clinically benefited.

**Table 2:** Metabolism evaluation criteria for immunotherapy response.

Criterion	CMR	PMR	SMD	PMD
EORTC (1999) (62)	complete resolution of ^18^F-FDG uptake within the tumor volume	SUV is reduced by at least 15% ~25% after 1 cycle of chemotherapy, and > 25% after more than one treatment cycle	not CMR, PMR, or PMD	SUV increase > 25%, visible increase in the extent of tumor ^18^F-FDG uptake (> 20% in the longest dimension), or appearance of new ^18^F-FDG uptake in metastatic lesions
PERCIST (2009) (63)	^18^F-FDG uptake completely disappeared	SULpeak decrease by ≥30% in the target lesions, and absolute drop in SUL by at least 0.8 SUL units	not CMR, PMR, or PMD	SULpeak in the target lesions increase ≥ 30%, with ≥ 0.8 SUL unit increase; 75% increase in TLG; new ^18^F-FDG-avid lesions that are typical of cancer and not related to treatment effect or infection
iPERCIST (2019) (64)	^18^F-FDG uptake completely disappeared	SULpeak decrease by ≥30% in the target lesions	not CMR, PMR, or PMD	SULpeak increase ≥ 30% or new ^18^F-FDG-avid lesions (UPMD) UPMD needs to be confirmed CPMD by a second PET after 4-8 weeks; if UPMD is followed by PMR or SMD, the bar is reset
imPERCIST5 (2019) (65)	^18^F-FDG uptake completely disappeared	SULpeak decrease by ≥30% in the target lesions, and absolute drop in SUL by at least 0.8 SUL units	not CMR, PMR, or PMD	SULpeak in the target lesions increase ≥ 30%, with ≥ 0.8 SUL unit increase in tumor SUVpeak; New lesions were included in the sum of SULpeak if they showed higher uptake than existing target lesions or if fewer than 5 target lesions were detected on the baseline scan

EORTC, European Organization for Research and Treatment of Cancer 1999 criteria; PERCIST, PET Response Criteria in Solid Tumors; imPERCIST5, immunotherapy-modified PERCIST, 5-lesion analysis; iPERCIST, immune PERCIST; CMR, complete metabolic response; PMR, partial metabolic response; SMD, stable metabolic disease; PMD, progressive metabolic disease; UPMD, unconfirmed progressive metabolic disease; CPMD, confirmed progressive metabolic disease; SUV, standard uptake value; SULpeak, peak standardized uptake values normalized for body lean body mass; SUL, standardized uptake value of lean body mass; TLG, total lesion glycolysis; SUVpeak, peak standardized uptake value.

The comparative studies of different response evaluation criteria are shown in **Table 3**. In short, the continuous adjustment of immunotherapy response evaluation criteria aims to guide immunotherapy management more precisely and effectively.

**Table 3:** Comparative studies on evaluation criteria of immunotherapy response.

Author/Year	Study/number	Histology	ICI Treatment	Criteria	Conclusion
Sachpekidis et al. (67). (2018)	prospective 41 patients	melanoma	ipilimumab	EORCT PERCIMT	The sensitivity of PERCIMT was significantly higher than EORTC, but the specificity was not significantly different.
Sachpekidis et al. (68). (2019)	prospective 16 patients	melanoma	ipilimumab	EORTC PERCIMT	PERCIMT shows more correct classification (15/16 patients) than EORTC (13/16 patients).
Goldfarb et al. (64). (2019)	retrospective 28 patients	NSCLC	nivolumab	iRECIST iPERCIST	iPERCIST can reclassify 39% of patients assessed by iRECIST.
Beer et al. (69). (2019)	prospective 42 patients	NSCLC	nivolumab, pembrolizumab or durvalumab	RECIST 1.1 iRECIST PERCIST	The three criteria are only moderately consistent, but there is no significant difference in the ability to assess PFS and OS after 12 months
Castello et al. (70). (2020)	prospective 35 patients	NSCLC	nivolumab or pembrolizumab	RECIST 1.1 imRECIST EORTC PERCIST imPERCIST PERCIMT	Fair agreement between imRECIST and EORTC, and PERCIST, and moderate for imRECIST and PERCIMT were detected. All criteria are significantly related to PFS, but only PERCIMT and imPERCIST are related to OS.
Dimitriou et al. (71). (2021)	retrospective 104 patients	melanoma	anti-PD-1 with or without anti-CTLA-4 treatment	RECIST EORTC	EORTC is better than RECIST in predicting progress, effectively assessing residual lesions on CT, and predicting long-term benefits.
Kitajima et al. (72). (2022)	retrospective 27 patients	melanoma	nivolumab or pembrolizumab	EORTC, PERCIST, imPERCIST	All the three FDG-PET criteria showed accuracy for response evaluation of ICI therapy and prediction of malignant melanoma patient prognosis.

RECIST, Response Evaluation Criteria in Solid Tumor; iRECIST, a modified RECIST 1.1 for immune-based therapeutics; EORTC, European Organization for Research and Treatment of Cancer 1999 criteria; PERCIMT, PET Response Evaluation Criteria for Immunotherapy; PERCIST, PET Response Criteria in Solid Tumors; iPERCIST, immune PERCIST; imPERCIST, immunotherapy-modified PERCIST; PFS, progression-free survival; OS, overall survival; NSCLC, non-small-cell lung cancer; PD-L1, programmed death-ligand 1; PD-1, programmed cell death protein-1; CTLA-4, cytotoxic T lymphocyte-associated protein 4.

### Prediction of prognosis

Since only a certain proportion of patients can benefit from ICIs therapy, how to conduct pretreatment assessments and identify eligible patients has important clinical significance. Up to now, some potentially prognostic biomarkers have been explored, including tumor PD-L1 expression, tumor mutation burden (TMB), microsatellite instability (MSI), gene expression profiles, gastrointestinal microbiome, and so on (73). Nevertheless, the values of these biomarkers remain controversial, and some biomarkers (such as TMB and MSI) require complex, expensive, and time-consuming analyses. Despite imperfection, PD-L1 expression is still the most commonly used biomarker in clinic, especially for NSCLC patients (74).

There have been a variety of studies committed to discovering the prognostic value of ^18^F-FDG PET/CT imaging on ICI treatment, but the results are inconsistent. The researches mainly focus on patients with NSCLC and melanoma, and the metabolic parameters include SUVmax, SUVmean, metabolic tumor volume (MTV), total MTV (tMTV), total lesion glycolysis (TLG), and so on (**Table 4**). Although SUVmax is the most commonly used metabolic parameter, its prognostic value may be controversial (81, 87). Some researchers have also advocated that SUVmean may be suggestive (83). Other studies support the prognostic value of MTV and TLG for immunotherapy, indicating that high MTV and TLG are associated with poor prognosis (78, 80). However, the multivariate analysis of a prospective study on nivolumab found no significant correlation between TLG and OS (88). In addition, tMTV provides a good indication of the total cancer burden (3). Seban et al. demonstrated that tMTV > 75 cm^3^ was associated with shorter OS and the absence of clinical disease benefit. They proposed a metabolic scoring system based on the derived neutrophil-to-lymphocyte ratio (dNLR) and tMTV, which stratified patients into three groups with different prognosis: poor prognosis (dNLR>3 and tMTV>75 cm^3^), moderate prognosis (dNLR>3 or tMTV>75 cm^3^) and good prognosis (dNLR ≤ 3 and tMTV ≤ 75 cm^3^) (77). However, Vekens et al. discovered that tMTV and TLG did not have a predictive effect (87).

**Table 4:** Prognosis predictive role of ^18^F-FDG PET/CT in immunotherapy.

Author/Year	Study/number	Histology	ICI treatment	Conclusion
Seban et al. (75) (2019)	retrospective, 55 patients	melanoma	anti-PD-1 IgG	Higher tMTV and BLR correlated with shorter survival
Ito et al. (76) (2019)	retrospective, 142 patients	melanoma	ipilimumab	wMTV was negatively correlated with OS
Seban et al. (77). (2020)	retrospective, 80 patients	NSCLC	anti-PD-1/PD-L1	tMTV > 75 cm^3^ was associated with shorter OS and absence of disease clinical benefit
Hashimoto et al. (78). (2020)	retrospective, 85 patients	NSCLC	pembrolizumab or nivolumab	TLG and MTV were negatively correlated with PFS and OS
Castello et al. (79). (2020)	prospective, 50 patients	NSCLC	nivolumab or pembrolizumab	High TLG and MTV were significantly associated with hyperprogression and MTV remained a negatively independent predictor for OS
Yamaguchi et al. (80). (2020)	retrospective, 48 patients	NSCLC	pembrolizumab	Higher MTV correlated with worse outcomes for patients with PD-L1 expression ≥50%
Polverari et al. (81). (2020)	retrospective, 57 patients	NSCLC	pembrolizumab	Patients with higher MTV and TLG values were more likely to have disease progression and poor response to immunotherapy.
Chardin et al. (82) (2020)	prospective, 75 patients	NSCLC	pembrolizumab or nivolumab	MTV and TLG were negatively correlated with OS and could reliably predict early treatment discontinuation
Seban et al. (83). (2020)	retrospective, 63 patients	NSCLC	pembrolizumab	Both high tMTV and high SUVmean were independent predictors for decreased PFS, and tMTV was also negatively correlated with OS.
Wong et al. (84). (2020)	retrospective, 90 patients	melanoma	ipilimumab, pembrolizumab or nivolumab	High pre-treatment SLR was associated with short PFS and OS
Seban et al. (85). (2020)	retrospective, 56 patients	melanoma	ipilimumab and/or pembrolizumab	In patients with mucosal melanoma, increased tumor SUVmax was correlated with shorter OS, while in patients with cutaneous melanoma, increased tMTV and BLR were independently correlated with shorter OS, PFS, and lower response
Dall’ Olio et al. (86). (2021)	retrospective, 34 patients	NSCLC	pembrolizumab	tMTV ≥ 75cm^3^ could be a prognostic predictor of inferior outcomes in patients with PD-L1 expression ≥ 50%
Vekens et al. (87). (2021)	retrospective, 30 patients	NSCLC	pembrolizumab	SUVmax was positively related to PFS. Clinical response and survival were independent of tMTV and TLG. Reduction of tMTV and TLG after 8 to 9 weeks of treatment was a better predictor of prolonged survival than RECIST 1.1.
Bauckneht et al. (88) (2021)	prospective, 45 patients	NSCLC	nivolumab	MTV was negatively related to OS
Awada et al. (89) (2021)	retrospective, 183 patients	melanoma	pembrolizumab	Elevated tMTV was associated with worse PFS and OS.
Gulturk et al. (90). (2021)	retrospective, 32 patients	RCC	nivolumab	Pre-treatment SUVmax was negatively related to PFS

SUVmax, maximum standardized uptake value; SUVmean, mean standardized uptake value; MTV, metabolic tumor volume; TLG, total lesion glycolysis; tMTV, total metabolic tumor volume; SLR, spleen-to-liver SUVmax ratio; BLR, bone marrow-to-liver SUVmax ratio; wMTV, whole-body metabolic tumor volume; PD-L1, programmed death-ligand 1; PD-1, programmed cell death protein-1; NSCLC, non-small-cell lung cancer; RCC, renal cell carcinoma; RECIST 1.1, Response Evaluation Criteria in Solid Tumors version 1.1; OS, overall survival; PFS, progression-free survival.

The main reasons for the inconsistent results may lie in the heterogeneity of patients included and the difference of treatment schemes in the study cohorts. The discrepancies between adopted end-point events and treatment response evaluation criteria may be other confounding issues. Further researches should be carried out to establish an ideal and universal method from the metabolism perspective for predicting the prognosis of pan-cancer, which would be validated in a large dataset.

In recent years, the rapid development of artificial intelligence has further promoted the application of ^18^F-FDG PET/CT radiomics, which extracts a large number of quantitative features from PET/CT images through automated and high-throughput methods, in the prognosis evaluation after surgery, chemoradiotherapy, targeted therapy or immunotherapy. Mu et al. (91) constructed a deep learning model based on PET/CT images, namely the EGFR-deep learning score (EGFR-DLS), to provide non-invasive decision support for targeted therapy or immunotherapy for patients with NSCLC. Moreover, Mu et al. (92) established a nomogram including multi-parameter PET/CT radiomic features, Eastern Cooperative Oncology Group (ECOG) score, and distant metastasis to predict the prognosis of patients with stage IIIB-IV NSCLC receiving ICIs therapy.

### Challenges and perspectives

With unprecedented advances in ICIs in cancer treatment, the value of ^18^F-FDG PET/CT has been especially emphasized. Nevertheless, several challenges associated with ^18^F-FDG PET/CT imaging need to be addressed to broaden its application in ICIs treatment. One major shortcoming is the lack of recognized guidelines to instruct the application of ^18^F-FDG PET/CT imaging in immunotherapy. On the other hand, although glucose metabolism parameters, such as SUVmax, have been shown to be significantly related to the immune characteristics of TME or prognosis, related studies were mostly retrospective, single-center and small-sample size, and there are controversies between the results of different studies. So, further prospective and large-sample cohort studies are still needed. Moreover, current studies mainly focus on NSCLC and melanoma. With the extensive development of ICIs treatment and the accumulation of cases, researches concentrating on other cancers can be investigated. Besides, there remains a challenging area of investigations on biological mechanisms of the association between TME and ^18^F-FDG PET/CT imaging, and basic and translational studies are encouraged to unravel the unknowns.

## New PET molecular probe imaging for ICIs treatment

**Figure 5:**
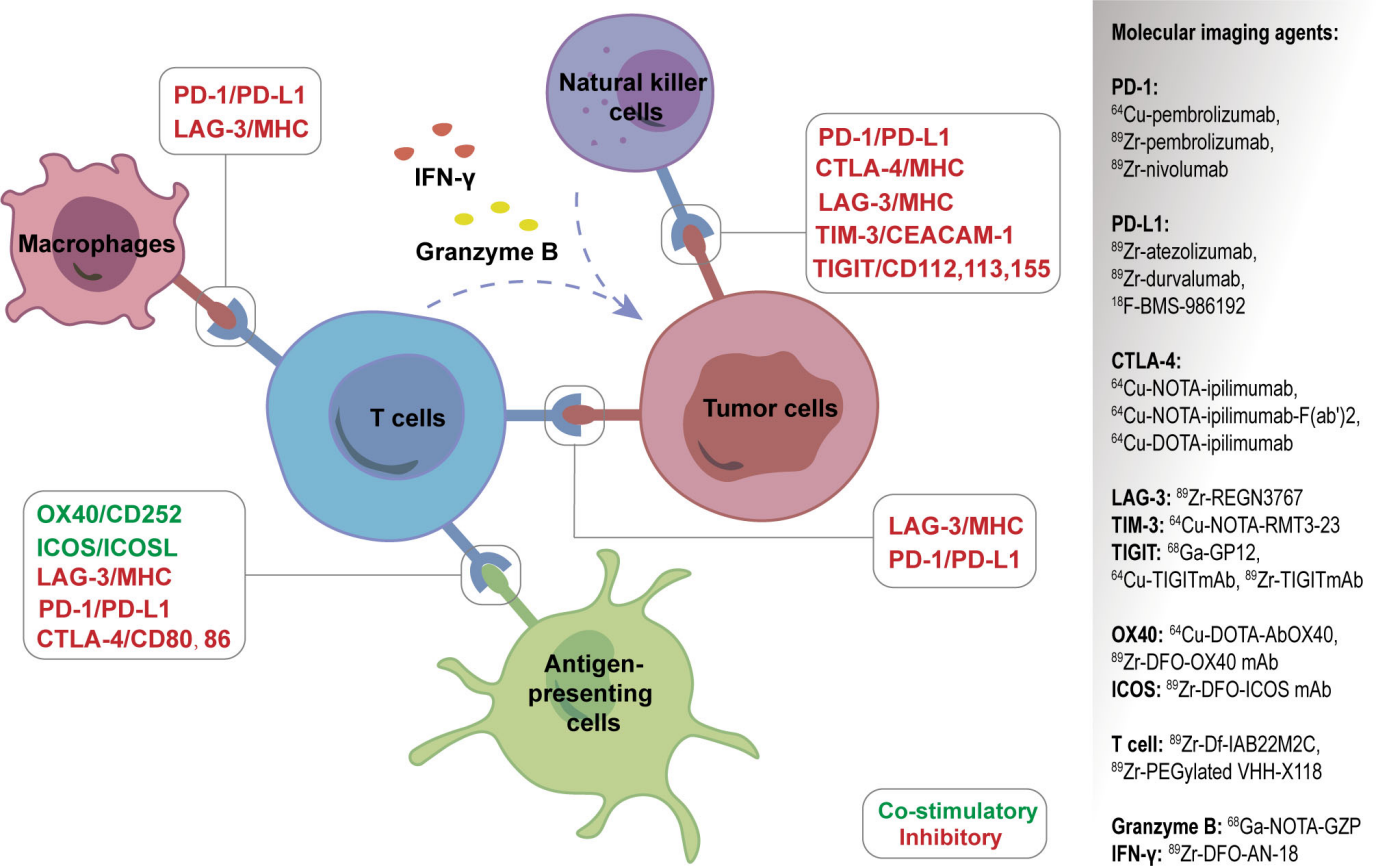
A series of new PET molecular probes targeting the compositions of tumor microenvironment (TME).

In addition to ^18^F-FDG, researchers are also committed to developing a series of new PET molecular probes targeting the compositions of TME, some of which have entered preclinical or clinical applications (**Figure 5**). The principles of these designs are mostly based on the specific binding of radiolabeled antibodies, peptides, or small molecules with the corresponding targets. The emergence of new molecular imaging agents is expected to provide more accurate means to obtain dynamic information about TME for promoting individualized treatment.

### PD-1/PD-L1 targeting

PD-L1 or PD-1 expression is reported to be related with prognosis after immunotherapy, and the detection of the two biomarkers mainly depends on immunohistochemistry (IHC) of biopsy or surgery materials. However, this invasive and snap-shot approach has some limitations, including the inability to reflect the heterogeneity and spatio-temporal dynamic expression of PD-L1 or PD-1 in tumor tissues, different antibody detection platforms and different thresholds leading to different results, and difficulties in obtaining histological specimens for some patients. Targeted molecular imaging can detect PD-L1 or PD-1 expression noninvasively and dynamically *in vivo* to compensate for the above shortcomings.

PD-1 imaging agents used in clinical trials include ^89^Zr-pembrolizumab, ^64^Cu-pembrolizumab, and ^89^Zr-nivolumab. The first-in-humans study of ^89^Zr-pembrolizumab in patients with advanced-stage NSCLC confirmed its safety and feasibility for immunotherapy. The findings indicated that patients with higher tumor uptake for ^89^Zr-pembrolizumab showed a tendency for better response to pembrolizumab (93). A later study showed that ^89^Zr-pembrolizumab uptake was positively associated with PFS and OS in melanoma and NSCLC patients (94).

PD-L1 targeting imaging agents mainly include ^89^Zr-atezolizumab, ^89^Zr-durvalumab, and ^18^F-BMS-986192, all of which have been in clinical trials (95). Researchers uncovered that ^89^Zr-atezolizumab uptake performed better than IHC or RNA sequencing-based predictive biomarkers in evaluating clinical responses for ICIs (96). ^89^Zr-atezolizumab is also reported to be helpful in identifying RCC patients who may benefit from anti-PD-1/PD-L1 therapy (97). Meanwhile, a number of preclinical studies on PD-L1-targeting molecular probes (including antibodies, peptides, and small molecules) using nuclear medicine, MRI, or optical imaging, have been documented (98, 99).

### CTLA-4 targeting

CTLA-4 is another well-known immunosuppressive checkpoint. It is expressed on T cells and binds with CD80/86 ligands on DCs with a high affinity to prevent uncontrolled expansion of activated T cells. Accordingly, the blockade of CTLA-4 with antibodies has been used in clinic as a promising option for cancer patients. CTLA-4 targeting imaging agents include ^64^Cu-NOTA-ipilimumab-F(ab’)2, ^64^Cu-NOTA-ipilimumab, and ^64^Cu-DOTA-ipilimumab (100, 101), and all of them have not yet entered clinical trials.

### Other immune checkpoints targeting

Apart from PD-1, PD-L1, and CTLA-4, several novel immune checkpoint molecules, both the inhibitory and stimulatory ones, have been discovered over the past decade (43). The former molecules include lymphocyte activation gene-3 (LAG-3), T cell immunoglobulin and mucin-domain containing-3 (TIM-3), T cell immunoglobulin and ITIM domain (TIGIT), sialic acid-binding immunoglobulin-like lectin 15 (Siglec-15), and V-domain Ig suppressor of T cell activation (VISTA), which express on a variety of immune cells and exhibit inhibitory roles in the context of malignancy.

The positive immune regulators, such as glucocorticoid-induced TNFR-related gene (GITR) and tumor necrosis factor receptor superfamily member 4 (OX40), are co-stimulatory molecules expressed on T cells. In addition, inducible T-cell co-stimulator (ICOS) is an indicator of T-cell-mediated immune response, and some animal experiments showed that ^89^Zr-DFO-ICOS mAb targeting ICOS could monitor immunotherapy response (102, 103). With the discovery of new immune checkpoints, molecular probes targeting these targets are emerging.

### T cell targeting

CD8^+^ TILs are an important feature reflecting TIME and significantly impact the tumorigenesis and development of tumors. Responders showed higher numbers of pre-existing CD8^+^ Teff cells within the tumor and at tumor margins prior to ICIs therapy (104). Therefore, targeted imaging of CD8^+^ T cells is of great significance for immunotherapy.

^89^Zr-Df-IAB22M2C, a new molecular probe targeting CD8^+^ TILs, is used to assess CD8^+^ TILs in tumors accurately. The first human trial of ^89^Zr-Df-IAB22M2C has proven its safety and validity in patients with solid malignancies (105). ^89^Zr-PEGylated VHH-X118 has also been confirmed to have the potential for CD8^+^ TIL targeting imaging (106). Iravani et al. demonstrated that ^18^F-FDG and other new imaging agents, such as those targeting CD8^+^ TILs or T cell function, can be used for PET/CT imaging to guide ICI treatment in the future (107). However, due to the complexity of lymphocyte subsets, CD8^+^ TILs imaging agents can only reflect part of the overall immune effect (108). Another problem with T cell targeting imaging is to determine the optimal timing of evaluation to reasonably reflect the activation degree of T cells.

### Secretory substance targeting

Secretory substances exist in the extracellular environment and participate in information transmission and effect exertion. Imaging targeting such substances, such as granzyme B and interferon-gamma (IFN-γ), has potential advantages in showing the immune treatment response. With regards to granzyme B targeted imaging, ^18^F-Ara-Gand and ^68^Ga-NOTA-GZP can reflect the activation of CD8^+^ T cells and help to distinguish between pseudoprogression and true progression (107). Another study showed that the detection of granzyme B with ^68^Ga-NOTA-GZP helps differentiate the responders from non-responders to immunotherapy (109). ^89^Zr-DFO-AN-18, a novel probe targeting IFN-γ, has been indicated to monitor the response to immunotherapy in mouse mammary tumors (110).

## Conclusion

In summary, an in-depth understanding of the underlying mechanisms of cancer immunotherapy is an important cornerstone for expanding the benefits of ICIs treatment to a larger cancer population. Hence, diagnostic approaches, especially PET/CT molecular imaging, should be vigorously developed to identify patients who might benefit from ICIs treatment. Concurrently, under the guidance of PET/CT molecular imaging, clinicians can shift the paradigm to improve the outcome of cancer patients, and even facilitate the development of novel therapeutic strategies to enhance therapeutic effectiveness. It is believed that with the extensive availability of standardized protocols, various affordable imaging agents, and user-friendly analysis platforms, PET/CT imaging will play a more important role in the era of immuno-oncology.

## Author contributions

Conceptualization, ML. Methodology, ML, YG, and CW. Material preparation, YG, CW, XC, LM, XZ, JC, and XL. Writing-original draft preparation, YG and CW. Writing-review and editing, ML and XC. Funding acquisition, ML. Supervision, ML. All authors contributed to the article and approved the submitted version.

## Funding

This study was supported by grants from the National Natural Science Foundation of China (82172052), Beijing Natural Science Foundation (Z210007), and National High-Level Hospital Clinical Research Funding (Interdepartmental Clinical Research Project of Peking University First Hospital) (2022CR34).

## Conflict of interest

The authors declare that the research was conducted in the absence of any commercial or financial relationships that could be construed as a potential conflict of interest.

## Publisher’s note

All claims expressed in this article are solely those of the authors and do not necessarily represent those of their affiliated organizations, or those of the publisher, the editors and the reviewers. Any product that may be evaluated in this article, or claim that may be made by its manufacturer, is not guaranteed or endorsed by the publisher.
